# Decreased ratios of matrix metalloproteinases to tissue-type inhibitors in cerebrospinal fluid in sporadic and hereditary cerebral amyloid angiopathy

**DOI:** 10.1186/s13195-023-01171-3

**Published:** 2023-01-30

**Authors:** Marc Vervuurt, Anna M. de Kort, Lieke Jäkel, Iris Kersten, Wilson F. Abdo, Floris H. B. M. Schreuder, Ingeborg Rasing, Gisela M. Terwindt, Marieke J. H. Wermer, Steven M. Greenberg, Catharina J. M. Klijn, H. Bea Kuiperij, Marcel M. Verbeek

**Affiliations:** 1 Department of Neurology, Cognition and Behaviour, Donders Institute for Brain, Radboud University Medical Center, P.O. Box 9101, 6500 HB , 830 TML Nijmegen, The Netherlands; 2 Department of Intensive Care Medicine, Radboud University Medical Center, Nijmegen, The Netherlands; 3 Department of Neurology, Leiden University Medical Center, Leiden, The Netherlands; 4 Department of Neurology, Massachusetts General Hospital, Boston, MA USA; 5 Department of Laboratory Medicine, Radboud University Medical Center, Nijmegen, The Netherlands

**Keywords:** Sporadic cerebral amyloid angiopathy, Hereditary cerebral amyloid angiopathy cerebrospinal fluid, Biomarkers, Matrix metalloproteinases, Tissue inhibitors of matrix metalloproteinases

## Abstract

**Background:**

To evaluate the potential of cerebrospinal fluid (CSF) levels of matrix metalloproteinases and tissue-type inhibitors (MMP; TIMP), and ratios of MMPs to TIMPs, to function as biomarkers for sporadic or hereditary cerebral amyloid angiopathy (CAA).

**Methods:**

CSF concentrations of the matrix metalloproteinases MMP-2, MMP-9 and MMP-14, as well as the tissue inhibitors of metalloproteinases TIMP-1, TIMP-2 and TIMP-3, were determined using immunoassays. These assays were applied to two, independent study groups of sporadic CAA (sCAA) (*n* = 28/43) and control subjects (*n* = 40/40), as well as to groups of pre-symptomatic (*n* = 11) and symptomatic hereditary Dutch-CAA (D-CAA) patients (*n* = 12), and age-matched controls (*n* = 22/28, respectively).

**Results:**

In the sCAA/control cohorts, inconsistent differences were found for individual MMPs and TIMPs, but MMP-2/TIMP-2 (discovery/validation: *p* = 0.004; *p* = 0.02) and MMP-14/TIMP-2 ratios (discovery/validation: *p* < 0.001; *p* = 0.04) were consistently decreased in sCAA, compared to controls. Moreover, MMP-14 was decreased in symptomatic D-CAA (*p* = 0.03), compared to controls. The MMP-14/TIMP-1 (*p* = 0.03) and MMP-14/TIMP-2 (*p* = 0.04) ratios were decreased in symptomatic D-CAA compared to controls and also compared to pre-symptomatic D-CAA (*p* = 0.004; *p* = 0.005, respectively).

**Conclusion:**

CSF MMP-2/TIMP-2 and MMP-14/TIMP-2 were consistently decreased in sCAA, compared to controls. Additionally, MMP-14/TIMP-2 levels were also decreased in symptomatic D-CAA, compared to both pre-symptomatic D-CAA and controls, and can therefore be considered a biomarker for sporadic and late-stage hereditary forms of CAA.

**Supplementary Information:**

The online version contains supplementary material available at 10.1186/s13195-023-01171-3.

## Background

Cerebral amyloid angiopathy (CAA) is a common cause of intracerebral haemorrhages and of vascular cognitive impairment. An estimated 23% of the general elderly population (over 55 years of age) has moderate-to-severe CAA pathology, with CAA prevalence highly correlating with increasing age [1]. This disease, driven by the deposition of amyloid beta peptides (Aβ) in the cerebral vasculature, induces a progressive, degenerative process compromising the cerebral vasculature to the point that it increases susceptibility for developing haemorrhages in the brain. The underlying amyloidotic pathology of CAA shows many parallels with that of Alzheimer’s disease (AD), and these diseases often coincide [1, 2]. CAA, however, also develops as an entity separate from AD, in sporadic (sCAA), as well as hereditary forms of CAA. One of these familial forms of CAA is caused by the E693Q mutation in the *APP* protein, known as Dutch-type CAA (D-CAA) [3, 4]. Whereas D-CAA presents with symptoms similar to sCAA, major differences include the earlier age of onset (20 years earlier before general onset in sCAA) and a more aggressive progression of pathology.

Current diagnostic guidelines (labelled the Boston Criteria 2.0) are used to diagnose clinical CAA using specific magnetic resonance imaging sequences, in combination with clinical symptoms. However, this method displays limited sensitivity and specificity with regard to diagnosing CAA and as such can only diagnose CAA with *probable* and *possible* likelihoods. These limitations are based on the fact that the Boston Criteria 2.0 mostly focus on late-stage pathological manifestations of CAA, including microbleeds and cortical superficial siderosis. *Definite* CAA diagnosis is only possible through post-mortem pathology studies [5]. Novel diagnostic tools and techniques to allow for earlier diagnosis of CAA are therefore desired, potentially including biochemical analyses of body fluids such as blood or cerebrospinal fluid (CSF). A major advantage of the latter approach is that CSF is in direct contact with the (pathological) vasculature of the central nervous system and would therefore reflect pathophysiological changes in the brain.

Many studies have implicated the potential involvement of matrix metalloproteinases (MMPs) in the development and progression of CAA [6, 7]. Increased proteolytic activity of MMPs is thought to progressively degrade the vascular extracellular matrix (ECM), compromising the integrity of the neurovascular unit and the blood–brain barrier [7–9]. Many MMPs are produced as pro-MMPs and either are activated intracellularly prior to secretion (e.g. MMP-2, MMP-9) or are secreted from the cell as pro-MMP, adhering to structures like the plasma membrane or the ECM, prior to activation (e.g. MMP-14) [10, 11]. Directly related to MMPs and the (patho)physiological processes in which they are implicated are tissue inhibitors of metalloproteinases (TIMPs) which modulate MMP activity through direct, stoichiometric inhibition of MMPs [12, 13]. Previous studies have proven that a large degree of multilateral interaction between all different (iso)forms of MMPs and TIMPs exists, illustrated by the complex web of many (in-)direct activation and inhibition interactions between MMPs and TIMPs [14–20].

Aβ40, the predominant Aβ form associated with CAA, has been shown to increase expression levels of MMP-2, MMP-9 and MMP-14 in cell and animal studies [21, 22]. Immunostaining of CAA brain tissue has revealed MMP-9 deposition in CAA-affected vessels in a severity-dependent manner [23, 24]. Moreover, a few studies found elevated expression levels of TIMP-3 in CAA [25, 26]. Analyses of MMP-2, MMP-9, TIMP-1 and TIMP-2 levels in CSF of AD patients, vascular dementia patients and controls revealed no differences in MMP or TIMP levels between these aforementioned groups. However, associations were discovered between the number of microbleeds and decreased CSF MMP-9, TIMP-1 and TIMP-2 levels in AD patients [27].

The aim of this study was to evaluate levels of MMP-2, MMP-9, MMP-14, TIMP-1, TIMP-2 and TIMP-3, and ratios between these MMPs and TIMPs (as a proxy of proteolytic activity), in CSF of CAA patients and control subjects, to determine potential mechanistic involvement and diagnostic functionality. We have done so by determining levels of these proteins in the CSF of groups of sCAA and D-CAA patients and control subjects through the use of immunometric assays.

### Methods

#### Patients and biological fluids

We constructed two independent groups of sCAA patients for analysis (a discovery and a validation group), as well as separate groups of pre-symptomatic and symptomatic D-CAA patients and respective age-matched controls (see Tables 1 and 3).

**Table 1:** Univariate analysis of MMP and TIMP biomarkers in CSF of sCAA patients and controls

	***n*** ** (CAA/CON)**	**sCAA**	**Controls**	***p*** **-value**	**Adjusted ** ***p*** **-value**
**Discovery cohort**					
**Demographics**					
Age (y)	28/40	72.3 (65.7–77.1)	64.2 (56.1–69.8)	*p* < 0.001 (***)^**‡**^	
Sex, M/F (% male)	28/40	19/9 (68%)	28/12 (70%)	*p* = 0.85 (ns)	
**CSF parameters**					
MMP-2 (ng/mL)	27/40	44.6 (36.7–48.1)	38.3 (30.1–46.3)	*p* = 0.02 (*)^**‡**^	*p* = 0.35 (ns)
MMP-9 (pg/mL)^	15/23	120.0 (85.7–208.0)	106.0 (89.3–306.0)	*p* = 0.46 (ns)	*p* = 0.46 (ns)
MMP-14 (ng/mL)^	28/40	1000 (699–1399)	1087 (771–1387)	*p* = 0.53 (ns)	*p* = 0.30 (ns)
TIMP-1 (ng/mL)	28/40	61.8 (53.6–74.0)	48.9 (36.4–61.9)	***p*** = 0.002 (**)	*p* = 0.08 (ns)
TIMP-2 (ng/mL)	27/40	56.2 (44.4–68.0)	37.2 (33.8–43.8)	***p*** < 0.001 (***)	***p*** < 0.001 (***)
TIMP-3 (pg/mL)	26/40	121.9 (88.2–157.9)	110.2 (88.2–157.9)	*p* = 1.00 (ns)	*p* = 0.97 (ns)
Total protein (mg/mL)	28/40	0.94 (0.85–1.05)	0.84 (0.75–0.97)	***p*** = 0.03 (*)	*p* = 0.19 (ns)
**Validation cohort**					
**Demographics**					
Age (y)	43/40	68.0 (60.0–75.0)	71.2 (64.5–74.1)	*p* = 0.30 (ns)^‡^	
Sex, M/F (% male)	43/40	22/21 (51.1%)	23/17 (57.5%)	*p* = 0.66 (ns)	
**CSF parameters**					
MMP-2 (ng/mL)	36/40	28.9 (24.5–32.8)	29.9 (25.9–37.9)	*p* = 0.33 (ns)	*p* = 0.55 (ns)
MMP-9 (pg/mL)^	27/40	930 (657–2345)	639 (399–1135)	***p*** = 0.02 (*)	*p* = 0.70 (ns)
MMP-14 (ng/mL)^	43/40	2440 (2054–3245)	2951 (2186–3828)	*p* = 0.15 (ns)	*p* = 0.16 (ns)
TIMP-1 (ng/mL)	43/36	41.5 (35.6–57.8)	40.3 (31.2–57.7)	*p* = 0.78 (ns)	*p* = 0.99 (ns)
TIMP-2 (ng/mL)	43/38	51.0 (44.2–57.9)	47.7 (42.6–64.1)	*p* = 0.17 (ns)	*p* = 0.32 (ns)
TIMP-3 (pg/mL)	37/37	123.2 (71.1–192.8)	88.8 (54.7–120.7)	***p*** = 0.03 (*)	***p*** = 0.02 (*)
Total protein (mg/mL)	43/40	0.88 (0.82–1.02)	0.89 (0.80–0.98)	*p* = 0.96 (ns)	*p* = 0.97 (ns)

Data are presented as median (IQR). *p*-values for CSF parameters are shown in two variants: (1) unadjusted and (2) adjusted for age of subjects. Nonsignificant (ns) *p* > 0.05, *p ≤ 0.05, **p < 0.01, ***p < 0.001. Significant differences are presented in bold and were tested using Mann–Whitney *U* (default, in case of non-parametric data) or Student’s *T*-test (‡, in case of parametric data). Group sizes differ per marker because of differences in available sample volumes. ^MMP-9 and MMP-14 levels were determined using different assays in discovery and validation studies. Absolute levels can therefore not be compared between studies

In the discovery cohort, we collected CSF samples of sCAA patients (*n* = 27) and control subjects (*n* = 40), all from the Radboud University Medical Center (Nijmegen, the Netherlands). sCAA patients were diagnosed using the modified Boston Criteria [5] and classified as probable (*n* = 21) or possible CAA (*n* = 2). Four patients who presented with both lobar and deep haemorrhages/microbleeds (*n* = 4; mixed CAA) were also included. The control group consisted of two major subsets. First are subjects (*n* = 30) who underwent a lumbar puncture in a routine diagnostic workflow to investigate the presentation of neurological symptoms or to exclude central nervous system (CNS) involvement of a systemic disease. These subjects did not suffer from the suspected neurological or neurodegenerative disease and were not found to suffer from known cognitive impairment, sepsis, CNS malignancies or a stroke (in the past 6 months). Second are subjects (*n* = 10) who underwent elective thoracoabdominal aortic aneurysm repair, for which an external lumbar drain was placed, as part of the standard of care, via which CSF was collected. These subjects also did not suffer from known cognitive impairment or a stroke (< 6 months) or from traumatic brain injury.

In the validation cohort, we were able to collect and obtain CSF samples from sCAA patients (*n* = 43) and control subjects (*n* = 40), according to the same inclusion criteria as mentioned in the discovery cohort. Samples of all control subjects were collected from the Radboud University Medical Center. Controls were included as described in the previous paragraph, including 4 patients undergoing thoracoabdominal aortic aneurysm repair patients. sCAA patient samples were acquired from either the Radboud University Medical Center (*n* = 12), the Leiden University Medical Center (Leiden, the Netherlands) (*n* = 9) or the Massachusetts General Hospital (Boston, MA, USA) (*n* = 22). Again, sCAA patients were diagnosed using the modified Boston Criteria and classified as definite CAA (*n* = 1), probable CAA with supporting pathology (*n* = 5) or probable CAA (*n* = 36). A patient who presented with both lobar and deep haemorrhages/microbleeds (mixed CAA) was also included (*n* = 1). Control groups were matched with regard to sex and age.

Additionally, we collected CSF samples from D-CAA patients. Patients were diagnosed through genetic sequencing, and these patients were further stratified according to whether patients were carriers of the *APP* c.2077G > C mutation and had no medical history of symptomatic (haemorrhagic) strokes (pre-symptomatic D-CAA; *n* = 11) or whether patients had already developed one or more symptomatic ICH(s), in combination with a c.2077G > C mutation, or at least one first-degree relative with a confirmed c.2077G > C mutation (symptomatic D-CAA; *n* = 12). Age- and sex-matched control groups were constructed for comparisons, according to the criteria as described above (*n* = 22 and *n* = 28 for pre-symptomatic D-CAA and symptomatic D-CAA patients, respectively). Again, controls were included in an identical manner as described previously, with 1 patient undergoing thoracoabdominal aortic aneurysm repair patients being included.

Every included participant underwent a lumbar puncture according to standard procedures, to collect CSF. CSF was collected in polypropylene tubes, centrifuged, aliquoted and subsequently stored at − 80 °C.

Local medical ethical committees of all participating centres (Nijmegen, Leiden, Boston) approved the use of CSF of patients, as well as controls. Additionally, informed consent was collected from all participants.

#### Biochemical analyses

ELISAs were used to quantify MMP-2, TIMP-1, TIMP-2 and TIMP-3 in CSF (all R&D DuoSets, R&D Systems, Minneapolis, MN, USA). MMP-9 was quantified using an ELLA Simple Plex MMP-9 automated immunoassay (ProteinSimple, San Jose, CA, USA) for the discovery cohort and a human MMP-9 ELISA (Raybiotech, Peachtree Corners, GA, USA) for the validation cohort. MMP-14 levels were determined using a human MMP-14 ELISA (Finetest, Wuhan, China). Levels of MMPs/TIMPs in CSF were determined at different dilutions: MMP-2 (6 × diluted), MMP-9 (2 × diluted ELLA; 4 × diluted ELISA), MMP-14 (batch-dependent dilutions: discovery group 4 × diluted, validation group 12 × diluted), TIMP-1 (100 × diluted), TIMP-2 (100 × diluted) and TIMP-3 (undiluted). Because available sample volumes per subject in a group differed, as well as the sample volume required per assay, sub-selections per biomarker had to be made of the CAA and control groups. Group sizes per biomarker can be found in Tables 1, 2, 3 and 4.

**Table 2:** Univariate analysis of MMP/TIMP ratios in CSF of sCAA patients and controls

MMP/TIMP ratio	*n* (sCAA/CON)	sCAA	Control	*p*-value	Adjusted *p*-value
**Discovery cohort**					
MMP-2/TIMP-2	27/40	0.81 (0.62–0.98)	0.98 (0.80–1.11)	*p* = 0.004 (**)^**‡**^	***p*** = 0.001 (**)
MMP-9/TIMP-2^	15/23	1.98 (1.70–2.86)	3.09 (2.73–10.0)	***p*** = 0.002 (**)	***p*** = 0.05 (*)
MMP-14/TIMP-1^	28/40	15.37 (12.26–20.65)	21.52 (16.55–25.58)	***p*** = 0.001 (***)	***p*** = 0.003 (**)
MMP-14/TIMP-2^	27/40	15.93 (11.77–22.90)	27.54 (19.64–37.57)	***p*** < 0.001 (***)	***p*** < 0.001 (***)
**Validation cohort**					
MMP-2/TIMP-2	33/39	0.57 (0.49–0.67)	0.65 (0.55–0.73)	***p*** = 0.02 (*)	***p*** = 0.02 (*)
MMP-2/TIMP-3	30/37	0.25 (0.19–0.412)	0.34 (0.24–0.64)	***p*** = 0.05 (*)	*p* = 0.07 (ns)
MMP-14/TIMP-2^	43/39	52.32 (38.48–65.57)	59.99 (50.71–75.34)	***p*** = 0.04 (*)	***p*** = 0.04 (*)
MMP-14/TIMP-3^	37/37	23.39 (17.10–35.43)	32.23 (22.67–57.58)	***p*** < 0.001 (***)	***p*** = 0.002 (**)

Data are presented as median (IQR). *p-*values for CSF parameters are shown in two variants: (1) unadjusted and (2) adjusted for age of subjects. Ratios which did not significantly differ (either adjusted or unadjusted) were not presented in this table. Nonsignificant (ns) *p* > 0.05, *p ≤ 0.05, **p < 0.01, *** *p* < 0.001. Significant differences are presented in bold and were tested using Mann–Whitney *U* (default, in case of non-parametric data) or Student’s *T*-test (‡, in case of parametric data). ^MMP-9 and MMP-14 levels were determined using different assays in discovery and validation studies. Absolute levels can therefore not be compared between studies. Group sizes differ per marker because of differences in available sample volumes. ^MMP-9 and MMP-14 levels were determined using different assays in discovery and validation studies. Absolute levels can therefore not be compared between studies

**Table 3:** Univariate analysis of MMP and TIMP biomarkers in CSF of (pre-)symptomatic D-CAA patients and controls

	**Pre-symptomatic D-CAA**					**Symptomatic D-CAA**				
	***n*** ** (CAA/CON)**	**D-CAA**	**Controls**	***p*** **-value**	**Adjusted ** ***p*** **-value**	***n*** ** (CAA/CON)**	**D-CAA**	**Controls**	***p*** **-value**	**Adjusted ** ***p*** **-value**
**Demographics**										
Age (y)	11/22	38.0 (31.0–52.0)	45.2 (36.5–51.3)	*p* = 0.61 (ns)^‡^	-	12/28	58.5 (52.8–65.8)	60.3 (51.6–65.9)	*p* = 0.95 (ns)^‡^	-
Sex, M/F (% male)	11/22	8/3 (73%)	11/11 (50%)	*p* = 0.28 (ns)	-	12/28	5/7 (41.7%)	17/11 (60.7%)	*p* = 0.32 (ns)	-
**CSF parameters**										
MMP-2 (ng/mL)	9/22	22.6 (17.8–25.6)	19.2 (13.2–21.7)	***p*** = 0.04 (*)	*p* = 0.18 (ns)	11/28	23.3 (19.2–25.8)	25.8 (20.4–30.4)	*p* = 0.29 (ns)	*p* = 0.34 (ns)
MMP-9 (pg/mL)	10/21	712 (534–1211)	634 (372–764)	*p* = 0.19 (ns)	*p* = 0.62 (ns)	12/28	1019 (657–1561)	693 (364–1071)	*p* = 0.17 (ns)	*p* = 0.66 (ns)
MMP-14 (ng/mL)	9/21	2.60 (2.20–3.07)	2.49 (1.95–2.79)	*p* = 0.31 (ns)	*p* = 0.98 (ns)	12/28	1.99 (1.76–2.47)	2.57 (1.99–3.58)	***p*** = 0.03 (*)	***p*** = 0.02 (*)
TIMP-1 (ng/mL)	10/22	30.6 (20.0–36.1)	26.0 (20.0–31.3)	*p* = 0.41 (ns)	*p* = 0.74 (ns)	12/28	40.2 (27.5–45.9)	35.2 (27.9–40.3)	*p* = 0.71 (ns)	*p* = 0.82 (ns)
TIMP-2 (ng/mL)	10/22	40.4 (34.2–48.1)	40.2 (34.1–46.5)	*p* = 0.67 (ns)	*p* = 0.66 (ns)	12/28	46.7 (40.2–52.3)	44.8 (40.7–50.4)	*p* = 0.68 (ns)^‡^	*p* = 0.71 (ns)
TIMP-3 (pg/mL)	10/17	87.9 (68.9–116.8)	50.5 (37.2 – 87.1)	*p* = 0.09 (ns)	*p* = 0.15 (ns)	12/26	91.3 (57.6–125.4)	87.3 (46.0–120.5)	*p* = 1.00 (ns) ^‡^	*p* = 0.97 (ns)
Total protein (mg/mL)	11/22	0.71 (0.65–0.84)	0.75 (0.64–0.80)	*p* = 0.67 (ns)	*p* = 0.47 (ns)	12/28	0.84 (0.74–0.94)	0.83 (0.68–0.92)	*p* = 0.81 (ns)	*p* = 0.90 (ns)

Data are presented as median (IQR). *p*-values for CSF parameters are shown in two variants: (1) unadjusted and (2) adjusted for age of subjects. Nonsignificant (ns) *p* > 0.05, *p ≤ 0.05. Significant differences are presented in bold and were tested using Mann–Whitney* U* (default, in case of non-parametric data) or Student’s *T*-test (‡, in case of parametric data). Group sizes differ per marker because of differences in available sample volumes

**Table 4:** Univariate analysis of MMP/TIMP ratios in CSF of (pre-)symptomatic D-CAA patients and controls

Pre-symptomatic D-CAA						Symptomatic D-CAA				
**Ratios MMP/TIMP**	***n*** ** (CAA/CON)**	**D-CAA**	**Controls**	***p*** **-value**	**Adjusted ** ***p*** **-value**	***n*** ** (CAA/CON)**	**D-CAA**	**Controls**	***p*** **-value**	**Adjusted ** ***p*** **-value**
MMP-14/TIMP-1	11/21	105.7 (69.8–159.9)	89.2 (72.7–107.0)	*p* = 0.84 (ns)	*p* = 0.60 (ns)	11/26	61.2 (51.0–71.4)	78.5 (54.7–103.8)	*p* = 0.07 (ns)	***p*** = 0.03 (*)
MMP-14/TIMP-2	11/21	60.1 (48.7–83.3)	58.2 (49.6–79.6)	*p* = 0.99 (ns)	*p* = 0.48 (ns)	12/27	43.4 (40.6–55.1)	54.1 (43.3–81.3)	***p*** = 0.04 (*)	***p*** = 0.02 (*)
MMP-14/TIMP-3	9/17	27.9 (24.2–34.7)	46.3 (30.8–61.3)	*p* = 0.05 (ns)	*p* = 0.18 (ns)	12/25	21.0 (17.3–32.1)	31.8 (22.6–47.8)	***p*** = 0.04 (*)	*p* = 0.08 (ns)

Data are presented as median (IQR). Ratios which did not significantly differ (either adjusted or unadjusted) were not presented in this table. *p-*values for CSF parameters are shown in two variants: (1) unadjusted and (2) adjusted for age of subjects. Nonsignificant (ns) *p* > 0.05, *p ≤ 0.05. Significant differences are presented in bold and were tested using Mann–Whitney *U* (all non-parametric data). Group sizes differ per marker because of differences in available sample volumes

Five pooled CSF samples were incorporated in all analyses to function as quality controls (QC), allowing for comparison between multiple plates and to correct for inter-assay variation, where necessary. All standards, controls and samples were assayed in duplicate in all assays. Additionally, total protein levels of all CSF samples were determined through the use of a Pierce™ BCA protein assay kit (Thermo Fisher Scientific, Waltham, MA, USA).

#### Statistical analyses

We analysed the generated data using IBM SPSS Statistics version 25.0.0.1 (IBM Corp., Armonk, USA) and Graphpad Prism version 5.03 (Graphpad Software, Inc., San Diego, USA). As an indirect measure of proteolytic activity, we have calculated the ratio between MMPs and TIMPs, by dividing the individual MMP levels by the TIMP levels per subject.

We used Shapiro–Wilk tests to assess the normality of data. Correlations between variables were determined by the use of Spearman correlation analyses. Differences between groups were determined using Student’s *T*-tests (for parametric data) or Mann–Whitney *U* tests (for non-parametric data). Also, sex differences between groups were determined using chi-squared tests. Outliers were determined using the Grubbs test for outliers. Corrections for the potential confounding (residual) influences of age were made using linear regression modelling. Test results were deemed statistically significant with a *p*-value ≤ 0.05.

### Results

#### sCAA patients

The median ages of sCAA and control subjects were different in the discovery cohort (*p* < 0.001), but not in the validation cohort (*p* = 0.12). sCAA and control groups in both discovery and validation cohorts were not significantly different in terms of sex of participants (*p* = 0.85 and *p* = 0.52 for discovery and validation cohorts, respectively). Total protein levels were significantly elevated in the sCAA discovery group (*p* = 0.03) compared to controls, an elevation that was lost after correction for residual age effects (*p* = 0.19). No significant differences in total protein levels were discovered in the validation cohort (*p* = 0.96) (Table 1).

**Fig. 1:**
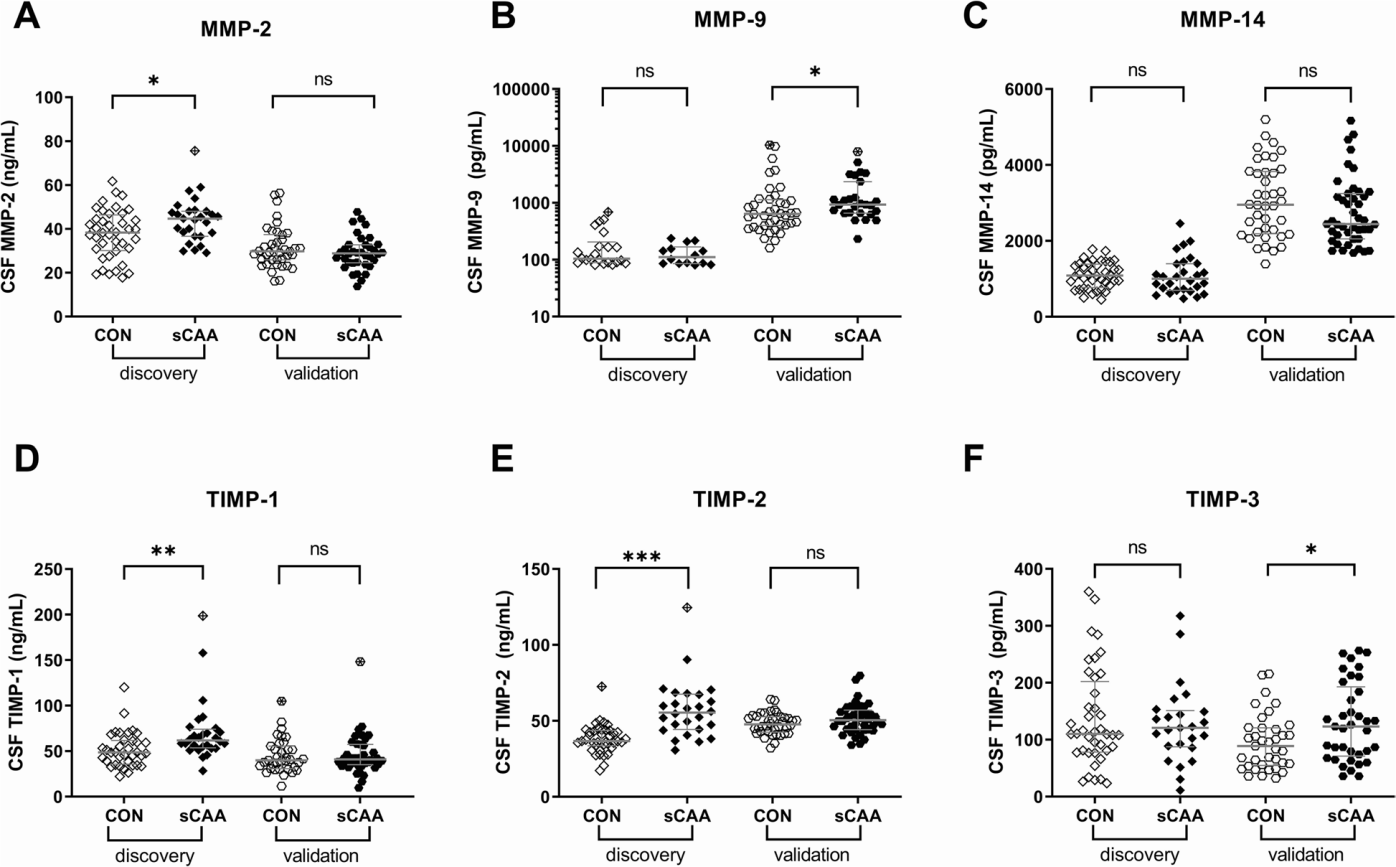
Univariate analysis of single MMP and TIMP biomarkers in CSF of sCAA patients and controls. In our discovery groups, significant elevations were discovered in the sCAA group for MMP-2 (Student’s *T*-test, *p* = 0.02), TIMP-1 (Mann–Whitney U, *p* = 0.002) and TIMP-2 (Mann–Whitney *U*, *p* ≤ 0.001). In the validation groups, significant elevations were discovered in the sCAA group for MMP-9 (Mann–Whitney *U*, *p* = 0.02) and TIMP-3 (Mann–Whitney *U*, *p* = 0.03). Crossed-out symbols signify statistically significant outliers (Grubbs test). Significance levels and accompanying *p*-values are of uncorrected comparisons. *p < 0.05, **p < 0.01, ***p < 0.001, ns *p* > 0.05

In the discovery cohort, elevated levels were discovered for MMP-2 (*p* = 0.02), TIMP-1 (*p* = 0.002) and TIMP-2 (*p* < 0.001), in sCAA patients compared to controls (Fig. 1). In the validation cohort, significant elevations in the sCAA relative to the control groups were found for MMP-9 (*p* = 0.02) and TIMP-3 (*p* = 0.03). After adjustment for age, the levels of TIMP-2 (discovery group; *p* < 0.001) and TIMP-3 (validation group; *p* = 0.02) remained significantly different between groups (Table 1).

**Fig. 2:**
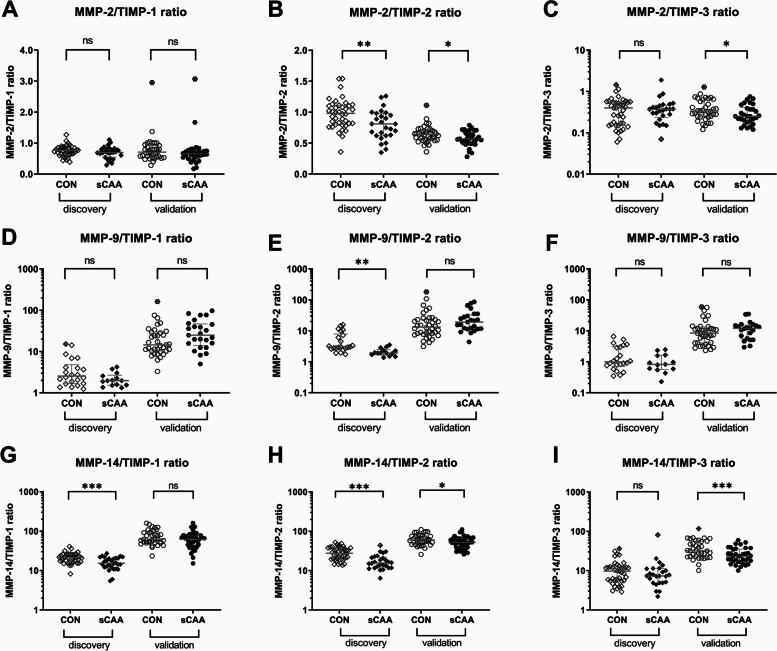
Univariate analysis of MMP/TIMP ratios in CSF of sCAA patients and controls. In our discovery groups, significant decreases were discovered in the sCAA group for MMP-2/TIMP-2 (Student’s *T*-test, *p* = 0.004), MMP-9/TIMP-2 (Mann–Whitney *U*, *p* = 0.002), MMP-14/TIMP-1 (Mann–Whitney *U*, *p* = 0.001) and MMP-14/TIMP-2 (Mann–Whitney *U*, *p* < 0.001). In the validation groups, significant decreases were discovered in the sCAA group for MMP-2/TIMP-2 (Mann–Whitney *U*, *p* = 0.02), MMP-2/TIMP-3 (Mann–Whitney *U*, *p* = 0.05), MMP-14/TIMP-2 (Mann–Whitney *U*, *p* = 0.04) and MMP-14/TIMP-3 (Mann–Whitney *U*, *p* < 0.001). Crossed-out symbols signify statistically significant outliers (Grubbs test). Significance levels and accompanying *p*-values are of uncorrected comparisons. *p < 0.05, **p < 0.01, ***p < 0.001, ns *p* > 0.05

As an indirect measure of the activity of MMP and TIMP levels, we assessed the MMP to TIMP ratios in CSF (Table 2 and Fig. 2). For the discovery cohort, this resulted in decreased ratios of MMP-2/TIMP-2 (*p* = 0.004), MMP-9/TIMP-2 (*p* = 0.002), MMP-14/TIMP-1 (*p* = 0.001) and MMP-14/TIMP-2 (*p* < 0.001), whereas for the validation cohort, this resulted in decreases in the ratios of MMP-2/TIMP-2 (*p* = 0.02), MMP-2/TIMP-3 (*p* = 0.05), MMP-14/TIMP-2 (*p* = 0.04) and MMP-14/TIMP-3 (*p* < 0.001). After the exclusion of outliers and adjustment for possible differences in age, decreased ratios were retained in the discovery cohort for MMP-2/TIMP-2 (*p* = 0.001), MMP-9/TIMP-2 (*p* = 0.05), MMP-14/TIMP-1 (*p* = 0.003) and MMP-14/TIMP-2 (*p* < 0.001). A similar analysis in the validation cohort yielded significant decreases in the MMP-2/TIMP-2 (*p* = 0.02), MMP-14/TIMP-2 (*p* = 0.04) and MMP-14/TIMP-3 (*p* = 0.002) ratios between sCAA and control groups.

We also executed correlation analyses, stratified to (absence of) CAA diagnosis, and separately for both discovery and validation groups (Supplementary material; Fig. A1). Moderate-to-strong significant correlations were discovered between MMP-2 and TIMP-2 in both sCAA patients and control subjects, in both cohorts (0.40 ≤ *ρ* ≤ 0.69) (Supplementary material; Fig. A2). Also, MMP-14 and TIMP-2 moderately correlated to each other (0.12 ≤ *ρ* ≤ 0.41). Also, several markers correlated with total protein levels, most prominently MMP-2 (0.35 ≤ *ρ* ≤ 0.63) and TIMP-2 (0.40 ≤ *ρ* ≤ 0.66).

#### D-CAA patients

**Fig. 3:**
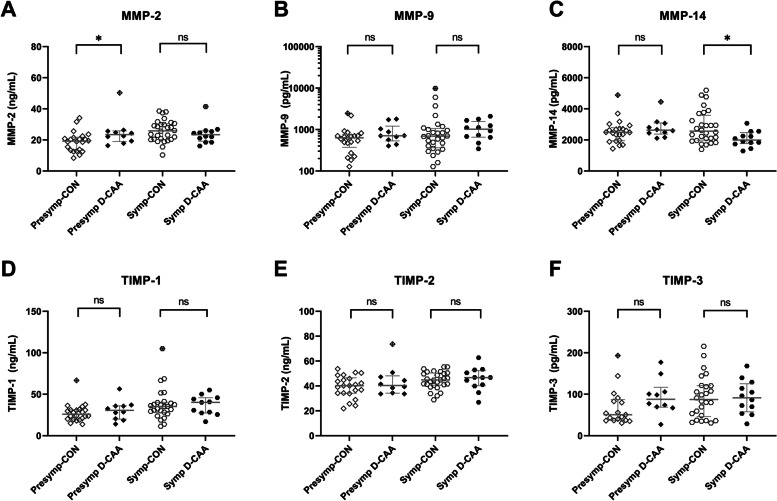
Univariate analysis of single MMP and TIMP biomarkers in CSF of (pre-) symptomatic D-CAA patients and controls. In the pre-symptomatic D-CAA group, significant elevations were discovered for MMP-2, compared to their control group (Mann–Whitney *U*, *p* = 0.04). In the symptomatic D-CAA group, significant decreases of MMP-14 levels were found, relative to their respective age-matched controls (Mann–Whitney *U*, *p* = 0.03). No other significant differences were discovered in biomarker levels between (pre-)symptomatic D-CAA patients and control subjects. Crossed-out symbols signify statistically significant outliers (Grubbs test). Significance levels and accompanying *p*-values are of uncorrected comparisons. *p < 0.05, ns *p* > 0.05

In the evaluation of the levels of MMPs and TIMPs in pre-symptomatic D-CAA patients versus those in controls, elevated levels were found for MMP-2 (*p* = 0.04), whereas symptomatic D-CAA patients presented with decreased MMP-14 levels compared to controls (*p* = 0.03) (Table 3 and Fig. 3). The exclusion of a significant TIMP-3 outlier in the control group resulted in a significant elevation of TIMP-3 levels in pre-symptomatic D-CAA (*p* = 0.09 without exclusion of the outlier, *p* = 0.04 with exclusion of the outlier). After correction for age, the significant difference of MMP-2 in pre-symptomatic D-CAA patients versus controls was lost (from *p* = 0.04 to p = 0.18). The observed significance of decreased MMP-14 in symptomatic D-CAA patients versus controls was retained after correction for age (from *p* = 0.03 to *p* = 0.02).

**Fig. 4:**
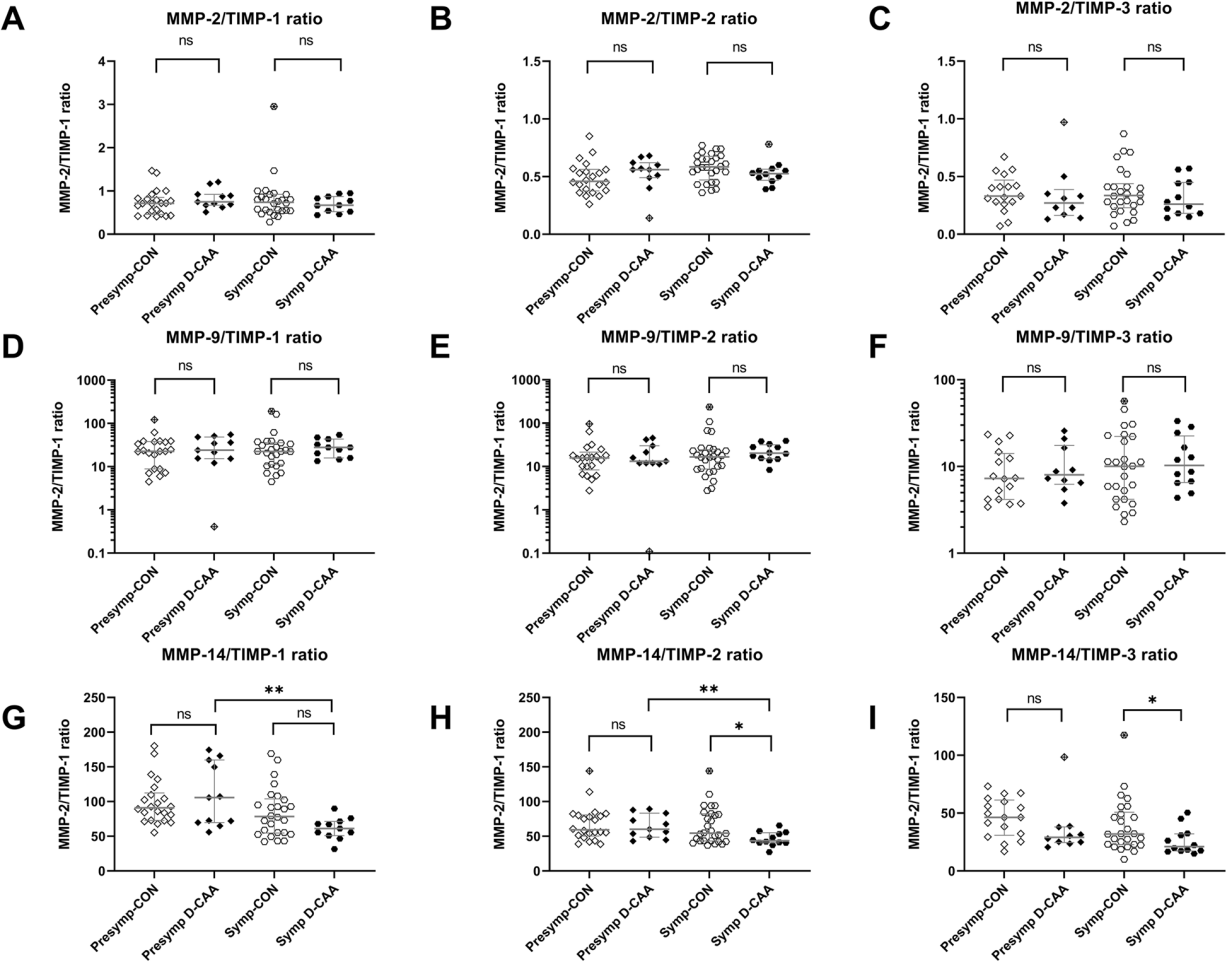
Univariate analysis of MMP/TIMP ratios in CSF of (pre-)symptomatic D-CAA patients and controls. In the pre-symptomatic D-CAA group, no significant differences were found in MMP/TIMP ratios between D-CAA patients and their age-matched controls. In the symptomatic D-CAA group, significant decreases of MMP-14/TIMP-2 ratios (Mann–Whitney *U*, *p* = 0.04) and MMP-14/TIMP-3 ratios (Mann–Whitney *U*, *p* = 0.04) were found, relative to their respective age-matched controls. Crossed-out symbols signify statistically significant outliers (Grubbs test). Significance levels and accompanying *p*-values are of uncorrected comparisons. *p < 0.05, ns *p* > 0.05

Ratios between MMPs and TIMPs showed no significant differences between pre-symptomatic D-CAA patients and control subjects after correction for age and the exclusion of outliers (Table 4 and Fig. 4). In symptomatic D-CAA patients versus control subjects, significant decreases of MMP-14/TIMP-2 (*p* = 0.04) and MMP-14/TIMP-3 (*p* = 0.04) were discovered. The MMP-14/TIMP-2 difference retained significance after correction for age and exclusion of outliers (from *p* = 0.04 to *p* = 0.02), whereas significance for differences in MMP-14/TIMP-3 ratio was lost (*p* = 0.08), and significant differences were discovered for MMP-14/TIMP-1 (*p* = 0.03). Whereas initially no difference was observed between pre-symptomatic D-CAA patients and control subjects in MMP-14/TIMP-3 ratio (*p* = 0.051), removal of the designated outlier in the control group for pre-symptomatic D-CAA patients did yield a substantial statistically significant difference (*p* = 0.008).

Comparisons of levels between pre-symptomatic and symptomatic D-CAA patients revealed decreased levels of MMP-14/TIMP-1 (*p* = 0.004) and MMP-14/TIMP-2 (*p* = 0.005) in symptomatic D-CAA patients. After the exclusion of potential outliers and correction for age, all differences were retained.

### Discussion

In this study, we evaluated the differences in CSF levels of MMP-2, MMP-9 and MMP-14, and TIMP-1, TIMP-2 and TIMP-3, in two independent groups of sCAA patients and control subjects and a group of D-CAA patients and controls. In sCAA, multiple differential protein levels were found, but were discovered to be inconsistent between both sCAA groups. These inconsistencies in findings again underscore the importance of replication of results of biomarker studies in independent cohorts. Consistent differences in both sCAA groups were discovered for the ratios of MMP-2/TIMP-2 and MMP-14/TIMP-2, which were decreased in sCAA compared to respective controls. Furthermore, elevated levels of MMP-2 were observed in pre-symptomatic D-CAA patients compared to controls and decreased MMP-14 levels in symptomatic D-CAA patients when compared to controls. Finally, ratios of MMP-14/TIMP-1 and MMP-14/TIMP-2 were decreased in symptomatic D-CAA patients, when compared to controls, and to pre-symptomatic D-CAA patients, after correction for age of subjects. CSF MMP-14/TIMP-2 thus appears to be consistently decreased in both sCAA (versus controls) and symptomatic D-CAA patients (versus pre-symptomatic D-CAA patients).

In case of differential levels of individual MMPs or TIMPs, we almost exclusively observed (at times inconsistent) elevations in sCAA compared to controls. This may be attributed to differences in composition between the groups. This, combined with the observed, robust reductions in ratios of MMPs to TIMPs could imply a relative increase in the expression of TIMPs compared to increases in the expression of MMPs, but these observations would need to be substantiated before definitive conclusions can be drawn.

**Fig. 5:**
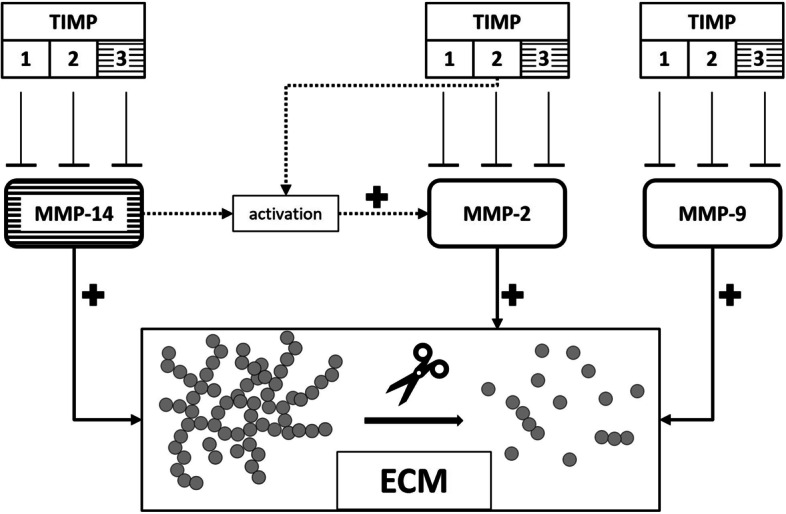
Schematic representation of mechanistic insights into MMP-2, MMP-9 and MMP-14, and, TIMP-1, TIMP-2 and TIMP-3 interactions. TIMP-1, TIMP-2 and TIMP-3 are capable of inhibition of MMP-2, MMP-9 and MMP-14. These MMPs are all capable of cleavage of extracellular matrix (ECM) proteins. Additionally, MMP-14 has a crucial role in the activation of proMMP-2 to MMP-2, a process in which TIMP-2 has an important facilitating function. MMP-2 and MMP-9 and TIMP-1 and TIMP-2 mostly appear as soluble factors, whereas MMP-14 and TIMP-3 are membrane-bound (dashed boxes). Solid lines signify direct, cleavage or inhibition relationships. Dashed lines signify indirect, facilitating roles in the activation of MMP-2

We did observe consistent differences in ratios between specific MMPs and TIMPs in both groups. Most remarkable were the findings that the MMP-2/TIMP-2 and MMP-14/TIMP-2 ratios were consistently decreased in sCAA, compared to controls. Moreover, the MMP-14/TIMP-2 ratio was also decreased in symptomatic D-CAA versus controls, and versus pre-symptomatic D-CAA patients. Interestingly, especially the interactions between MMP-2, MMP-14 and TIMP-2 have been studied extensively in the past: The respective genes *Mmp14* and *Timp2* are found to be remarkably co-expressed in mice [28]. Mechanistically, MMP-14 is known to be an activator of MMP-2 by cleavage of pro-MMP-2, a process in which TIMP-2 acts as a catalyst, by bridging MMP-14 and pro-MMP-2 [10, 29]. In this process, extracellular MMP-14 in homo-dimer/multimer complex can be inhibited by TIMP-2, after which the resultant configuration is capable of binding pro-MMP-2. MMP-14 then cleaves off the pro-domain of MMP-2, releasing activated MMP-2 and TIMP-2. Additionally, both MMP-14-TIMP-2 and MMP-14-TIMP-2-pro-MMP-2 complexes are found inside human cells [30]. A simplified model of these known mechanistic interactions between MMPs and TIMPs and their mutual effect on the ECM is visible in Fig. 5. The strong degree of interconnection between MMP-2, MMP-14 and TIMP-2 was reflected in this study, as the soluble MMP-2 and TIMP-2 proteins moderately-to-strongly correlated with each other (Supplementary material; Fig. A2). On the other hand, MMP-14 correlated weakly with TIMP-2 levels, possibly due to the (mostly) membrane-bound nature of MMP-14, in contrast with the soluble nature of TIMP-2.

Previous research has demonstrated that elevations in MMP or TIMP levels in CAA are related to the deposition of Aβ in the vasculature, as observed in CAA pathology [31–36]. Also, the aforementioned research has shown that many MMPs are capable of cleaving Aβ, that—in turn—Aβ is capable of increasing the expression of Aβ-cleaving MMPs and that the expressions of MMPs and TIMPs are highly correlated. Logically, Aβ peptides in the vasculature could induce elevated expression of Aβ-cleaving MMPs, which in turn stimulates expression of TIMPs to counteract increased proteolytic activity by MMPs: an increase in expression of the former would result in an increase of expression of the latter. Decreased MMP/TIMP ratios could imply reduced proteolytic MMP activity, which could in turn result in reduced degradation of vascular Aβ, progressing CAA pathology. However, MMP and TIMP levels, and ratios, might also be affected by CAA pathology. Whether these observed, robust changes in MMP/TIMP ratios are a cause or an effect of CAA pathology cannot yet be determined, but this topic is beyond the scope of the experimental design of this study.

Previous studies have investigated MMP-2, MMP-9, MMP-10, and TIMP-1 and TIMP-2 levels in CSF of AD patients with and without lobar microbleeds, which can be considered hallmarks of CAA [27]. However, the co-occurrence of AD in these patients can significantly confound results when extrapolating these results to CAA pathology. Additionally, CSF MMP-2 has been shown to be associated with parenchymal amyloid deposition [37]. MMP activity studies using gelatin zymography have shown MMP-9 activity to be elevated in CSF of patients with vascular dementia, but not in patients with AD, when compared to controls [38]. MMP-2 activity levels were found to be reduced in CSF of subcortical ischemic vascular disease patients, when compared to controls [39]. So although there have been some studies into (singular) associations of MMPs and TIMPs with CAA, no studies have evaluated CSF levels of multiple MMPs and TIMPs in well-characterized CAA subjects as we have done (to our knowledge).

The strengths of our study include the construction of cohorts of sCAA patients and controls, which were very well characterized using clinical data, CSF parameters and MRI. The use of two independent cohorts contributes to the robustness of the findings and decreases the possibility of false positive or negative results. Also, as far as we know, the group sizes in this study are large in comparison to previous biomarker studies in sCAA, strengthening the robustness of the findings. Additionally, the constructed, unique set of (pre-symptomatic) D-CAA patients and respective controls enabled the study of biomarker levels in relation to different stages of CAA pathology.

Limitations include a small number of included subjects for the D-CAA groups, reducing the power of the analyses performed on these groups, which makes drawing definitive conclusions more difficult. Also, due to technical reasons, different MMP-9 assays were used between the discovery and the validation groups, and the MMP-14 assay required batch-dependent dilutions, which were different between both aforementioned groups. As a result, absolute MMP-9 and MMP-14 levels cannot be compared between these substudies. An extra limitation of the use of the aforementioned ELISAs for determining MMP levels in CSF is the fact that said ELISAs are unable to differentiate between pro- and active forms of MMPs, and care should be taken in directly correlating MMP levels to MMP activity. Furthermore, only the validation cohort was age- and sex-matched, whereas in the discovery cohort, significant differences were observed for age of subjects and total protein levels. Especially the significant difference in age between sCAA patients and control subjects in the discovery group can therefore be considered to have contributed to the inconsistencies in results observed between the discovery and validation cohorts. However, no correlations between age of subjects and examined biomarkers were discovered (Supplementary Fig. 1). For future studies, special attention should be paid to matching study groups and cohorts to minimize the influence of potential confounders (especially sex and age).

## Conclusion

In conclusion, our study showed that the MMP-2/TIMP-2 and MMP-14/TIMP-2 ratios were decreased in sCAA patients compared to control subjects. The MMP-14/TIMP-2 ratio was also decreased in symptomatic D-CAA. Because of the similarities in the phenotypical nature of sCAA and symptomatic D-CAA, this biomarker seems an interesting hallmark of changes in the balance between MMP activity and TIMP inhibition, associated with the progression of early-stage sCAA to late-stage sCAA, and similarly, from pre-symptomatic to symptomatic D-CAA.

## Supplementary Information

**Additional file 1: Figs. A1 and A2.**

## Data Availability

The datasets used and/or analysed during the current study are available from the corresponding author on reasonable request.

## Abbreviations

Aβ40: Amyloid-β40
Aβ42: Amyloid-β42
AD: Alzheimer’s disease
BCA: Bicinchoninic acid assay
(s)CAA: (Sporadic) cerebral amyloid angiopathy
CNS: Central nervous system
CSF: Cerebrospinal fluid
D-CAA: Hereditary Dutch CAA (E693Q)
ECM: Extracellular matrix
ELISA: Enzyme-linked immunosorbent assay
MMP: Matrix metalloproteinase
QC: Quality control
TIMP: Tissue inhibitor of metalloproteinase
