# Supplementary material for: Decreased ratios of matrix metalloproteinases to tissue-type inhibitors in cerebrospinal fluid in sporadic and hereditary cerebral amyloid angiopathy
Source: Alzheimers Res Ther. 2023 Jan 30;15:26. doi: 10.1186/s13195-023-01171-3 (PMC9885599; doi:10.1186/s13195-023-01171-3)
Supplement: Supplementary file 1 — Additional file 1: Figs. A1 and A2. [file 13195_2023_1171_MOESM1_ESM.docx]

**Additional file 1**

*
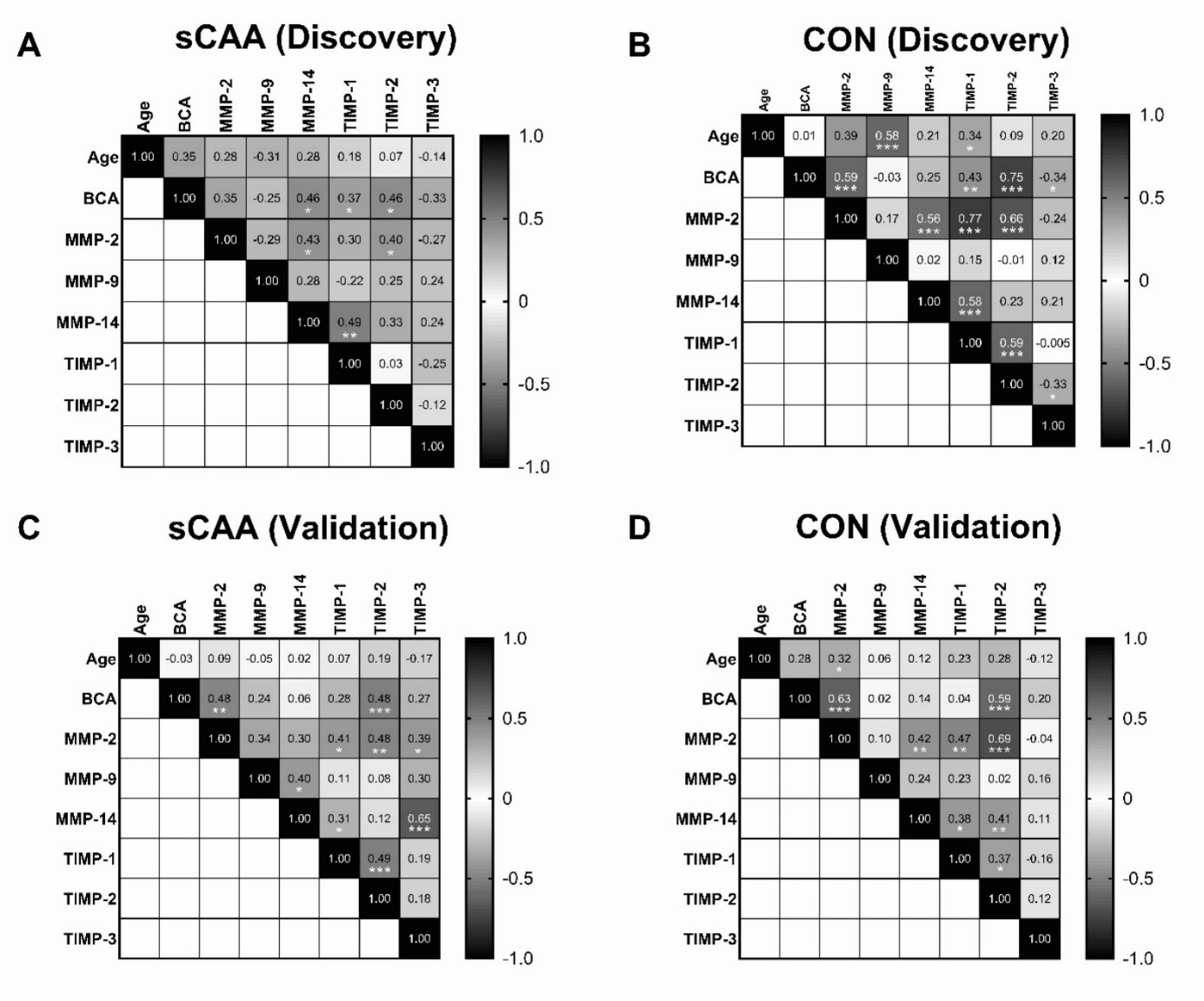
*

**Figure A1: Correlation heatmaps of variables in sCAA patients (sCAA) and control subjects (CON) in the discovery and validation cohorts.** Heatmaps of Spearman r values are shown for all correlations between the variables age, and CSF levels of total protein, MMP-2, MMP-9, MMP-14, TIMP-1, TIMP-2, and TIMP-3 in quadrants. Separate correlation quadrants are shown for the sCAA groups (A-C) and for the control groups (B-D) of both the discovery and the validation cohort, respectively. Grayscales (legend on right-hand side of each quadrant) signify correlation coefficients between 1.0 (black) and -1.0 (white). Significance of correlations are signified as follows: * p < 0.05, ** p < 0.01, *** p < 0.001.


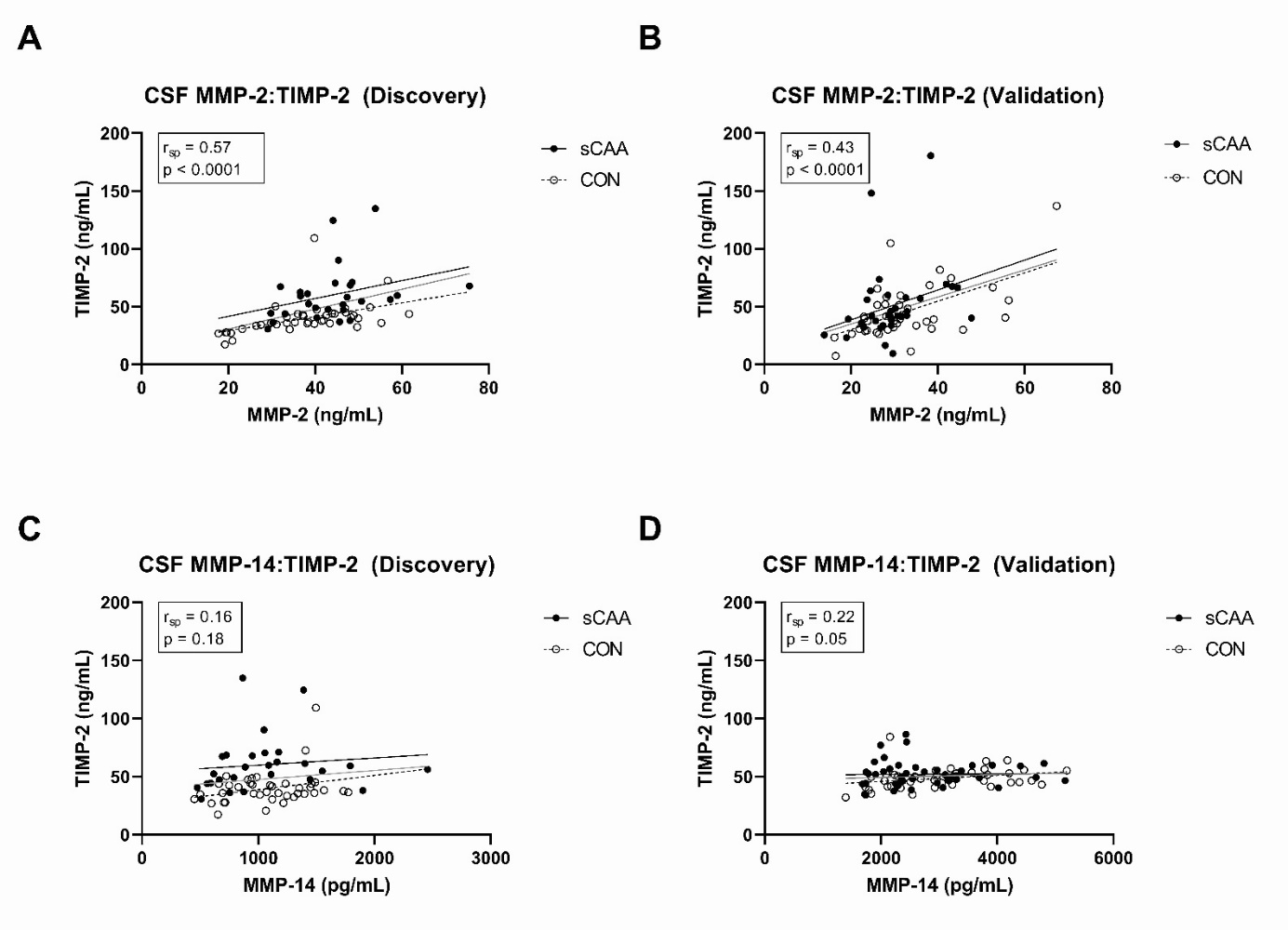


**Figure A2: Correlation scatterplots of MMP-2/TIMP-2 and MMP-14/TIMP-2 levels in CSF of sCAA and control subjects in the discovery and validation cohorts.** Spearman correlation analyses between MMP-2 and TIMP-2, and between MMP-14 and TIMP-2 in de discovery and validation cohorts. Correlation coefficients shown were calculated for combined sCAA and control groups. MMP-2 and TIMP-2 levels were found to be highly significantly correlated in sCAA patients and control subjects (r_sp_ ≥ 0.43 and r_sp_ = 0.57; both p < 0.001). On the other hand, MMP-14 and TIMP-2 levels were found to be not correlated, or only minutely correlated (r_sp_ = 0.16 and r_sp_ = 0.22; p = 0.18 and p = 0.05 respectively).
